# Two Cases of Cytomegalovirus Colitis During the Treatment of Immune Checkpoint Inhibitor-Associated Colitis

**DOI:** 10.7759/cureus.63308

**Published:** 2024-06-27

**Authors:** Masaya Iwamuro, Takehiro Tanaka, Go Makimoto, Eiki Ichihara, Sakiko Hiraoka

**Affiliations:** 1 Department of Gastroenterology and Hepatology, Okayama University Hospital, Okayama, JPN; 2 Department of Pathology, Okayama University Hospital, Okayama, JPN; 3 Department of Allergy and Respiratory Medicine, Okayama University Hospital, Okayama, JPN; 4 Department of Gastroenterology and Hepatology, Okayama University Graduate School of Medicine, Dentistry, and Pharmaceutical Sciences, Okayama, JPN

**Keywords:** immune-related adverse events, immune-checkpoint inhibitor, cytomegalovirus infection, colonoscopy, colitis

## Abstract

Herein, we outlined two case reports of patients who developed cytomegalovirus colitis following the initiation of corticosteroid therapy for colitis as a result of immune-related adverse events (irAEs). For both patients, endoscopic findings were similar to those observed for patients with irAE colitis but were devoid of the characteristic features associated with cytomegalovirus colitis, including punched-out ulcers. Given the therapeutic disparities between these two conditions, it is imperative to distinguish between these conditions in clinical practice. When addressing exacerbations or refractory manifestations of irAE-associated colitis, clinicians should remain vigilant with regard to the potential for cytomegalovirus infection, even in the absence of punched-out ulcers in the colorectum.

## Introduction

Immune-related adverse events (irAEs) refer to side effects that can occur as a result of immunotherapy with immune checkpoint inhibitors (ICIs) such as anti-cytotoxic T-lymphocyte-associated protein 4 (CTLA-4), anti-programmed cell death protein 1 (PD-1), and anti-programmed death-ligand 1 (PD-L1) drugs [1,2]. Colitis, one of the most common irAEs detected in cancer patients undergoing treatment with ICIs, involves inflammation of the colorectum driven by an abnormal immune response triggered by immunotherapeutic drugs. Cytomegalovirus infection can develop in the colorectum during the management of irAE colitis, particularly after the administration of corticosteroids [3]. Both irAE colitis and cytomegalovirus colitis manifest in the colorectum and exhibit comparable symptoms, including diarrhea, bloody stools, and fever. Being able to distinguish between these two conditions in clinical practice is critical because their treatment approaches differ. While irAE colitis may be managed by temporarily stopping or adjusting immunotherapy treatment and by administering corticosteroids to suppress the immune response, cytomegalovirus colitis typically requires antiviral medications to target the cytomegalovirus infection. In particular, clinicians need to consider that the intensification of immunosuppressive therapy in cases of irAE colitis, including corticosteroids, can exacerbate the risk of cytomegalovirus colitis in the presence of cytomegalovirus infections. Therefore, the early diagnosis of cytomegalovirus colitis and the initiation of appropriate antiviral therapy are crucial in the management of irAE colitis. However, comprehensive reports on cytomegalovirus colitis following irAE colitis are sparse, with merely nine cases documented to date.

We recently observed two cases in which cytomegalovirus colitis concurrently manifested during treatment for irAE colitis. Both cases presented with endoscopic findings resembling those of irAE colitis instead of the classical features of cytomegalovirus colitis, such as punched-out ulcers. These two cases highlight an intriguing clinical scenario encountered during the management of irAE colitis. We also review previously reported similar cases.

## Case presentation

Case 1

**Figure 1:**
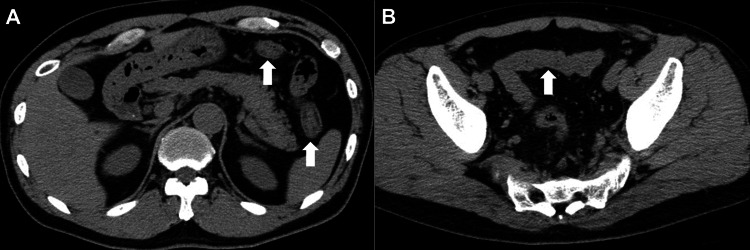
Computed tomography images acquired from Case 1. Thickening of the wall from the transverse colon (A, arrows) to the rectum (B, arrow).

A 61-year-old Japanese man was diagnosed with a malignant melanoma of the left great toe. The patient underwent amputation of the left great toe and was diagnosed with melanoma (T3bN2aM0; stage IIIc). He was administered adjuvant nivolumab monotherapy, an anti-PD-1 antibody. However, in week 37 post-surgery, two black pigmented lesions appeared on the left lower leg; a skin biopsy confirmed the diagnosis of recurrent metastatic melanoma. Treatment with a combination of ipilimumab, an anti-CTLA-4 antibody, and nivolumab was commenced in week 41 post-surgery. Six days after the initiation of combination therapy, the patient developed diarrhea at a frequency of 5-10 times per day. Suspecting irAE colitis, a computed tomography (CT) scan was performed, which revealed a thickening of the wall from the transverse colon to the rectum (Figure 1).

**Figure 2:**
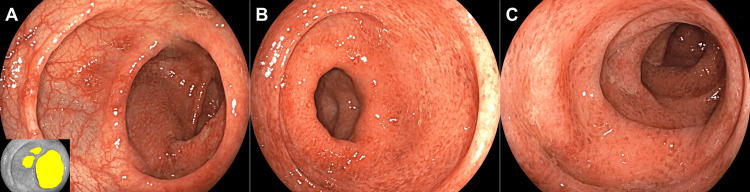
Colonoscopy images acquired from Case 1. Initial colonoscopy reveals a reddened and edematous mucosa with reduced vascular transparency from the transverse colon to the rectum. In the hepatic flexure, areas of reddened mucosa were partially observed (A, highlighted in yellow in the inset image). In the transverse colon (B) and sigmoid colon (C), reddened mucosa with reduced vascular transparency was diffusely observed.

Blood tests revealed a white blood cell count of 4,380/μL and a serum C-reactive protein level of 0.22 mg/dL; the test for cytomegalovirus antigenemia was negative. A colonoscopy revealed a reddened and edematous mucosa with reduced vascular transparency from the transverse colon to the rectum (Figure 2).

**Figure 3:**
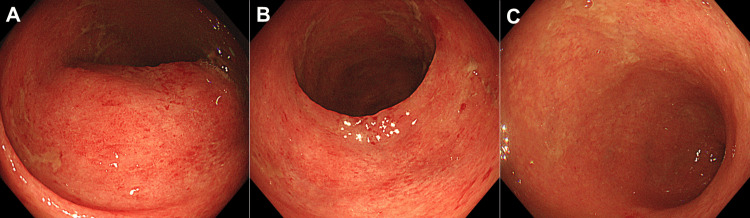
Colonoscopy images of cytomegalovirus colitis acquired from Case 1. Five days after the initiation of intravenous methylprednisolone, a reddened and edematous mucosa with reduced vascular transparency is diffusely observed on colonoscopy. Ulcer formation is absent. A: transverse colon; B: sigmoid colon; C: rectum.

**Figure 4:**
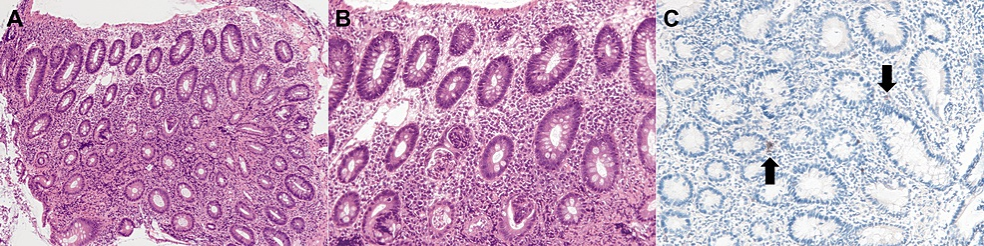
Pathological images acquired from Case 1. Hematoxylin and eosin stain show dense infiltration of lymphocytes and neutrophils in the lamina propria of the colon (A, ×4; B, ×10). The immunostaining of endoscopic biopsy specimens is positive for cytomegalovirus (C, arrows, ×10).

A biopsy of the colorectum revealed dense lymphocyte and neutrophil infiltration. This finding was consistent with colitis symptoms and supported the diagnosis of irAE colitis. Immunostaining further revealed that the colorectal mucosa was negative for cytomegalovirus. Treatment was initiated with oral prednisolone at a dose of 30 mg/day. The frequency of diarrhea decreased by 50%. However, 12 days after the initiation of prednisolone treatment, the diarrhea worsened, occasionally accompanied by bloody stools. In addition, the patient developed a fever (38.0°C). Considering the possibility of the exacerbation of irAE colitis, the patient was hospitalized (16 days after the initiation of prednisolone treatment) and began intravenous methylprednisolone at a dose of 80 mg/day. Blood tests after patient admission revealed a white blood cell count of 2,350/μL and a serum C-reactive protein level of 1.33 mg/dL. Despite the administration of intravenous methylprednisolone, there was no improvement in symptoms. A colonoscopy, performed five days after the initiation of intravenous methylprednisolone, revealed a reddened and edematous mucosa with reduced vascular transparency (Figure 3), similar to our initial findings. Colonoscopy did not detect any evidence of ulcer formation. However, endoscopic biopsy specimens were positive for cytomegalovirus, as determined by immunostaining (Figure 4). No inclusion bodies were observed under microscopic examination.

Additionally, the cytomegalovirus antigenemia test result was positive (pp65 antigen test, 1 positive cell/50,000 leukocytes). Based on these results, the worsening of symptoms was attributed to cytomegalovirus colitis. Oral administration of valganciclovir was initiated, while methylprednisolone was gradually tapered off. These treatments led to an improvement in diarrhea, bloody stools, and fever. The cytomegalovirus antigenemia test result was negative on the seventh day after the initiation of valganciclovir treatment. The patient experienced no recurrence of diarrhea for the subsequent five months. During this period, antitumor treatment was paused; however, an increase in the size of the left inguinal lymph nodes, attributed to metastasis of malignant melanoma, was observed. Consequently, resumption of treatment with nivolumab monotherapy is planned.

Case 2

**Figure 5:**
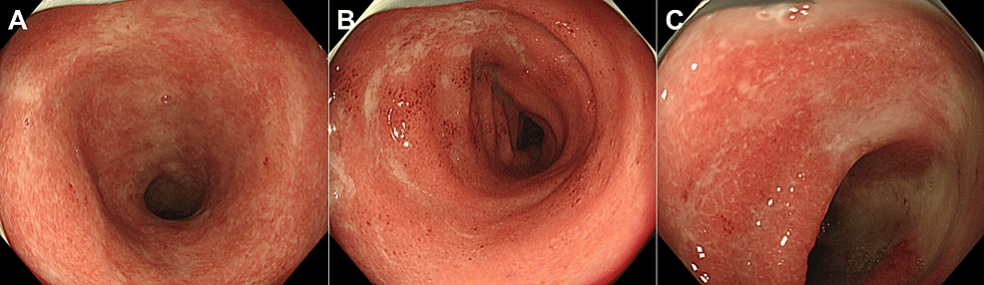
Colonoscopy images acquired from Case 2. A diffuse edematous and coarse mucosa is observed in the rectum up to the descending colon. A: descending colon; B: sigmoid colon; C: rectum.

A 75-year-old Japanese man was diagnosed with pulmonary adenocarcinoma (cT1bN0M1b, stage IVA) with rib and cerebellar metastases. The patient underwent CyberKnife treatment for primary lung lesions and rib metastases. Subsequently, monotherapy with pembrolizumab, an anti-PD-1 antibody, was initiated. On the eighth day after the initial dose of pembrolizumab, the patient developed watery diarrhea, occurring 7-8 times per day. The volume of watery stools temporarily decreased following the administration of corticosteroids. However, there was no complete resolution, and the diarrhea symptoms worsened. The patient was hospitalized 15 days after starting pembrolizumab treatment due to a lack of improvement. Blood tests revealed a leukocyte count of 6,980/μL and a serum C-reactive protein level of 2.62 mg/dL; the cytomegalovirus antigenemia test was negative. A colonoscopy performed on the day of admission revealed a diffuse edematous, and coarse mucosa extending from the rectum to the descending colon without apparent erosions or ulcers (Figure 5).

**Figure 6:**
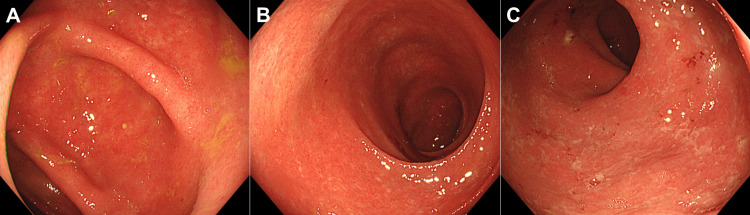
Colonoscopy images of cytomegalovirus colitis acquired from Case 2. Areas of a reddish and rough mucosa with decreased vascular transparency are seen in the ascending colon up to the rectum. A: ascending colon; B: sigmoid colon; C: rectum.

A biopsy revealed dense inflammatory cell infiltration and cryptitis in the colonic mucosa, indicative of irAE colitis. Immunostaining did not detect cytomegalovirus infection in the colorectal mucosa. On admission, treatment for irAE colitis was initiated with intravenous methylprednisolone at a dose of 60 mg/day. Despite the initiation of methylprednisolone, the watery diarrhea persisted. After 15 days of methylprednisolone therapy, the cytomegalovirus antigenemia test result was positive (pp65 antigen test, 69 positive cells/50,000 leukocytes), prompting the initiation of intravenous ganciclovir treatment. Five days after initiating ganciclovir treatment, the cytomegalovirus antigenemia test remained positive (9 positive cells/50,000 leukocytes). A colonoscopy performed 11 days after the initiation of ganciclovir therapy revealed areas of reddish, rough mucosa with decreased vascular transparency, extending from the ascending colon to the rectum (Figure 6).

**Figure 7:**
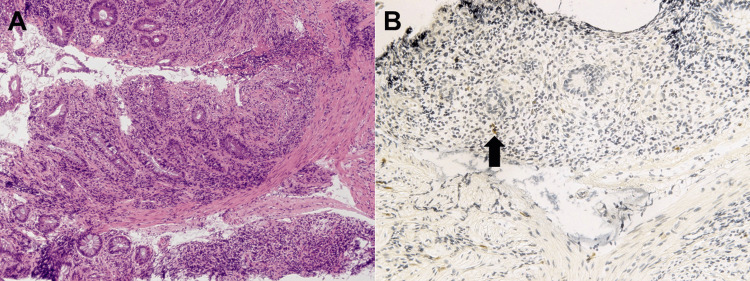
Pathological images acquired from Case 2. Hematoxylin and eosin staining reveals the infiltration of inflammatory cells, predominantly monocytes, in the rectal mucosa (A, ×4). Biopsies show a small number of cytomegalovirus-positive cells (B, arrow, ×10).

In the rectum, the mucosa was friable and exhibited white plaques. No ulcers were observed in the colorectum. A biopsy further revealed a small number of cytomegalovirus-positive cells in the rectal mucosa (Figure 7). Inclusion bodies were not observed in the biopsy specimens. The cytomegalovirus antigenemia test was negative 16 days after the initiation of ganciclovir treatment. The diarrhea symptoms improved, and the patient was discharged 21 days after commencing ganciclovir therapy. The patient has been under observation without antitumor treatment for the subsequent four months.

## Discussion

Cytomegalovirus colitis has been recognized as a significant complication in patients with ulcerative colitis. Punched-out ulcers represent classical endoscopic features of cytomegalovirus colitis [4,5] and are characterized by well-defined circular or oval areas of tissue loss or erosion and resemble holes punched out from the lining of the gastrointestinal tract. Punched-out ulcers typically exhibit distinct borders and vary in size. Previous studies have reported frequencies of punched-out ulcers ranging from 52-80% in patients with ulcerative colitis and cytomegalovirus colitis [6,7]. In contrast, the two cases of cytomegalovirus colitis presented here exhibited no apparent ulcer formation; instead, our findings resembled those of the initial colonoscopic images (irAE colitis).

Detailed reports of cytomegalovirus colitis complicating irAE colitis are scarce, with only nine cases documented to date [8-15]. Among these nine cases, there were six male and three female patients, with a median age of 66 years (range: 32-77 years). The primary diseases observed in these nine patients included melanoma (n = 5), lung adenocarcinoma (n = 2), small cell lung cancer (n = 1), and nasopharyngeal carcinoma (n = 1). The ICIs administered were nivolumab (n = 2), nivolumab and ipilimumab (n = 2), ipilimumab (n = 1), nivolumab followed by ipilimumab (n = 1), tislelizumab (n = 1), atezolizumab (n = 1), and toripalimab (n = 1). In all cases, cytomegalovirus colitis occurred following the administration of corticosteroids for irAE colitis. In one case, endoscopy images were not provided. Thus, the presence of ulcers could not be confirmed. In the remaining patients, punched-out ulcers were observed in four cases but not in the remaining four cases. The mechanism behind the absence of ulcer formation in some cases remains unclear, and further case accumulation and studies are needed to elucidate this phenomenon. Considering the endoscopic presentations of our two cases, along with the cases reported previously, it is important to highlight that the emergence of cytomegalovirus colitis during treatment for irAE enteritis may not always be associated with the presence of punched-out ulcers in the colorectum.

As mentioned earlier and evidenced by gastrointestinal perforation in two instances of cytomegalovirus colitis complicated by irAE colitis, the prompt initiation of antiviral therapy is essential for the treatment of cytomegalovirus infection [8, 14]. A retrospective study of 41 patients with irAE colitis identified five patients (12%) who did not respond to standard treatments, such as corticosteroids and infliximab [16]. Notably, cytomegalovirus was detected in all cases that were refractory to therapy. The European Society of Clinical Microbiology and Infectious Diseases (ESCMID) Study Group for Infections in Compromised Hosts (ESGICH) consensus document states that ICIs do not independently increase the risk of infection [17]. However, they can cause a range of irAEs that often necessitate additional immunosuppressive therapy, such as corticosteroids and/or infliximab, thereby elevating the infection risk. Consequently, when managing the exacerbations or refractoriness of colitis symptoms in patients with irAEs following immunosuppressive therapy, clinicians should consider the possibility of cytomegalovirus infection. This emphasizes the need for blood tests and histopathological analysis of endoscopic biopsies to detect cytomegalovirus. Although guidelines recommend testing for cytomegalovirus at the onset of Grade 2 or higher colitis after the use of ICIs [18], they do not address cytomegalovirus testing following the initiation of immunosuppressive therapy. In our personal opinion, cytomegalovirus testing should be considered in the following situations: i) when colitis symptoms worsen after starting immunosuppressive therapy, ii) when there is no improvement in colitis symptoms after seven days of immunosuppressive therapy, and iii) when there is a need to consider intensifying immunosuppressive therapy in cases of irAE colitis.

## Conclusions

We encountered two patients who developed a cytomegalovirus infection in the colorectum while undergoing treatment for irAE colitis. Notably, colonoscopy revealed endoscopic features resembling those of irAEs, highlighting the significance of considering cytomegalovirus involvement in cases presenting with persistent or worsening colitis symptoms, regardless of the presence of punched-out ulcers.
